# Prevalence of trachoma in the Kayes region of Mali eight years after stopping mass drug administration

**DOI:** 10.1371/journal.pntd.0006289

**Published:** 2018-02-12

**Authors:** Lamine Traoré, Benoit Dembele, Modibo Keita, Steven D Reid, Mahamadou Dembéle, Bréhima Mariko, Famolo Coulibaly, Whitney Goldman, Dramane Traoré, Daouda Coulibaly, Boubacar Guindo, Joseph J Amon, Marily Knieriemen, Yaobi Zhang

**Affiliations:** 1 Programme National de la Santé Oculaire, Ministère de la Santé, Bamako, Mali; 2 Mali Office, Helen Keller International, Bamako, Mali; 3 Headquarters, Helen Keller International, New York, New York, United States of America; 4 District Sanitaire de Kita, Direction Régionale de la Santé de Kayes, Kita, Mali; 5 Regional Office for Africa, Helen Keller International, Dakar, Senegal; University of California San Francisco, UNITED STATES

## Abstract

**Background:**

In 2009, three years after stopping mass treatment with azithromycin, a trachoma impact survey in four health districts in the Kayes region of Mali found a prevalence of trachomatous inflammation—follicular (TF) among children aged 1 to 9 years of >5% and a trachomatous trichiasis (TT) prevalence within the general population (≥1-year-old) of <1%. As a result, the government’s national trachoma program expanded trichiasis surgery and related activities required to achieve trachoma elimination.

**Methodology/Principal findings:**

In 2015, to assess progress towards elimination, a follow-up impact survey was conducted in the Kayes, Kéniéba, Nioro and Yélimané health districts. The survey used district level two-stage cluster random sampling methodology with 20 clusters of 30 households in each evaluation unit. Subjects were eligible for examination if they were ≥1 year. TF and TT cases were identified and confirmed by experienced ophthalmologists. In total 14,159 people were enumerated and 11,620 (82%) were examined. TF prevalence (95% confidence interval (CI)) was 0.5% (0.3–1%) in Kayes, 0.8% (0.4–1.7%) in Kéniéba, 0.2% (0–0.9%) in Nioro and 0.3% (0.1–1%) in Yélimané. TT prevalence (95% CI) was 0.04% (0–0.25%) in Kayes, 0.29% (0.11–0.6%) in Kéniéba, 0.04% (0–0.25%) in Nioro and 0.07% (0–0.27%) in Yélimané.

**Conclusions/Significance:**

Eight years after stopping MDA and intensifying trichiasis surgery outreach campaigns, all four districts reached the TF elimination threshold of <5% and three of four districts reached the TT elimination threshold of <0.1%.

## Author summary

Trachoma is a leading infectious cause of preventable blindness worldwide. The World Health Organization (WHO) recommends that endemic states implement the SAFE strategy (surgery for trichiasis, antibiotic treatment, facial cleanliness and environmental improvement) to achieve trachoma elimination by the year 2020. Mali has implemented this strategy from 2002. In the Kayes region in southwest Mali, the districts of Kayes, Kéniéba, Nioro and Yélimané stopped antibiotic treatment in 2006 after three rounds of mass drug administration (MDA). In 2009 an assessment was conducted and found that the prevalence of active trachoma (TF) among children aged 1–9 years and trichiasis (TT) prevalence in adults were above the elimination thresholds in these districts. An intensified effort to expand trichiasis surgery and related interventions, including community-based behavior change communication on facial cleanliness and environmental improvement activities using women’s groups, community health workers, and radio. A follow-up survey was conducted in 2015 in the four districts to assess whether one more round of mass treatment was needed. The results showed that TF prevalence in all four health districts decreased to below the 5% elimination threshold and three of four districts achieved the TT elimination threshold of <0.1%.

## Introduction

Trachoma is an eye disease caused by infection with *Chlamydia trachomatis* [1, 2]. Trachoma is transmitted by direct contact with discharge from the eyes or nose of infected individuals; repeated, untreated, infections can result in blindness [3]. In 2017, the World Health Organization (WHO) estimated that blinding trachoma is endemic in 41 countries worldwide which are known to require interventions, including 26 of the 46 sub-Saharan African countries, representing 90.1% of the population living in endemic areas globally that need interventions [3]. Trachoma afflicts the most disadvantaged people in the world, causing disability, loss of independence and is a barrier to social and economic development [4]. Worldwide, approximately 1.4 million people suffer from trachoma-related visual impairment; of whom 0.45 million suffer from irreversible blindness [5].

Surveys conducted in Mali from the 1980s showed high prevalence of active trachoma in children below 10 years in many regions, often exceeding 25% [6, 7]. National mapping of trachoma conducted in 1996–1997 found very high prevalence of trachomatous inflammation—follicular (TF) among children aged 1–9 years with an average prevalence, outside of the capital Bamako, of 34.9% [7]. The Kayes region, in the southwest of the country, was among the most affected, with a TF prevalence of 42.5% among children aged 1–9 years and trachomatous trichiasis (TT) prevalence among the adult female population of 3.3% [7].

In 1996, the WHO created the Alliance for the Global Elimination of Trachoma by 2020 (GET 2020) [8, 9]. The WHO recommended endemic states to implement the SAFE strategy to achieve the trachoma elimination by the year 2020 [1, 2, 10]. Among the four components of the SAFE strategy, “S” stands for surgery to correct trichiasis and preserve sight; “A” for mass antibiotic treatment, using azithromycin and tetracycline eye ointment to clear the infection; “F” for facial cleanliness to reduce the presence of ocular infections and nasal discharge; and “E” for environmental improvement to improve household access to water and latrines for better sanitation and hygiene [1, 2, 10, 11].

WHO also defined a threshold for the implementation of mass drug administration (MDA) of a TF prevalence in a health district ≥10% among children aged 1–9 years [12–14]. Mali’s National Blindness Prevention Program (PNSO) implemented the SAFE strategy gradually in high-prevalence regions nationwide. Many activities were implemented in the Kayes region between 2002–2006, including performing 3,371 trichiasis surgeries, distributing azithromycin (donated by Pfizer) and tetracycline ointment during MDA on an annual basis between 2004–2006, building 2,190 household latrines, rehabilitating 81 wells for clean water, launching 16 community-based radio stations for disseminating health messages, and training 16 local associations in trachoma information, education, and communication [15].

As recommended by the WHO 2006 guidelines [14], an impact assessment of these activities was conducted in 2009 in seven health districts in the Kayes region three years after the MDA was stopped in 2006. The TF prevalence among children aged 1–9 years in the Kayes, Kéniéba, Nioro and Yélimané health districts was 5.3%, 7.1%, 8.7% and 6.5%, respectively, while the TT prevalence within the general population was 0.75%, 0.78%, 0.59% and 0.20%, respectively [15]. Instead of conducting further surveys and conducting sub-district level (or community by community) MDA as suggested by the WHO guidelines [14], the PNSO decided not to resume MDA but to strengthen TT surgery campaign in these four health districts and implement facial cleanliness and environmental improvement activities via training for women’s groups and community health workers, and trachoma-centered radio messages. In 2014, through a technical consultation on trachoma surveillance by the WHO Strategic and Technical Advisory Group on NTDs, the WHO issued the new standard operating procedures recommending that in districts where there is a TF prevalence of between 5 and 9.9% in children aged between 1 and 9 years the F and E components of the SAFE strategy should continue as well as an additional round of MDA at the district level (including antibiotic MDA throughout the entire district). According to the recommendations from this technical consultation, this should be followed by a repeat impact assessment [16].

In light of these recommendations, the Mali PNSO decided to conduct an impact survey in these four health districts in 2015 before making the decision to implement another round of MDA. In this paper, we present the results of the impact survey and discuss the next steps for trachoma elimination in these four districts.

## Methods

### Study site

The Kayes region is Mali’s largest administrative region and is located in the western part of the country, bordered on the east by the Koulikoro region, west by the Republic of Senegal, north by the Islamic Republic of Mauritania, and south by the Republic of Guinea. The population in 2015 was estimated at 2,445,000 projected from the 2009 General Population and Housing Census, Mali [17], with an average density of 18.54 residents/km^2^. Nearly the entire population (90.6%) of the region lives in rural areas. The region is ethnically diverse with the population composed primarily of Khassonké, Malinké and Soninké people. The climate is arid in the north and semi-arid in the south, with average annual rainfall of 516 mm in the region. The region lacks adequate access to clean water, with an average of 190 inhabitants per clean water point [18]. The Kayes region is divided into eight health districts. The four districts included in the study, Kayes, Kéniéba, Nioro and Yélimané, are located respectively at west, south and north part of the Kayes region.

### Study type, sample size, and selection of clusters

To assess TF and TT prevalence in the four districts included in the study, a cross-sectional survey was conducted using two-stage, cluster random sampling methodology [16, 19]. Based upon this guidance, Kéniéba, Nioro and Yélimané health districts were defined as individual “evaluation units”, while in the more populous Kayes district, two separate evaluation units were defined.

Assuming a TF prevalence of 4%, a precision estimate of ±2%, an alpha of 5%, and a design effect of 2.7, a sample size of 996 children aged 1–9 years for each evaluation unit was calculated. An extra 12% was added to the sample size to adjust for refusals and this gave rise to an expected sample size of 1132 children aged 1–9 years per evaluation unit. The sample size for adults (≥15 years) was not calculated as all adult household members present at the selected households were to be examined for TT and other trachoma signs.

Twenty clusters were selected per evaluation unit using the probability proportional to size sampling strategy, from a complete list of villages, and population, in each health district [14, 20]. The sampling interval was obtained by dividing the evaluation unit’s population by the number of clusters. A random number between one and the sampling interval was then selected and the community whose cumulative population equaled this number was the first community selected. The 19 remaining clusters were then selected by adding the sampling interval on a successive basis [20, 21].

### Household selection and survey

The survey was implemented by teams, composed of an examiner (ophthalmic medical assistants) and a data collector. Each team was responsible for 10 clusters, with two teams per district. Teams were supervised by a senior ophthalmologist, with expertise in screening of TF and TT cases, responsible for confirming all detected cases [16].

With the assistance of village authorities, field teams prepared a complete list of households in selected villages, broken down into segments of five households. The last segment could include four or six households. Six segments were then chosen randomly, for a total of 30 households per village [22]. The households chosen were not replaced when the residents were absent or refused to participate. Before leaving the village on the survey day, survey teams revisited households where residents were absent at the time of the teams’ first visit.

All household members aged ≥1 year were examined for signs of trachoma. The eye examination was conducted using a magnifier visor (2.5x magnification) in natural light or with a flashlight. When a case of trichiasis was found, the examiner was advised to evert the eyelid to determine and record the presence or absence of conjunctival scarring. If such scarring was present, or if the eyelid could not be everted, this was considered a TT case. Information was recorded on whether surgery was offered to individuals with TT in order to estimate the number of TT cases unknown to the health system [16]. The WHO simplified grading system was used to classify the signs of trachoma [23].

In addition to looking for signs of trachoma, examiners also checked for discharge from the eyes and/or nose to assess facial cleanliness among all children aged 1–9 years. Children aged 5–14 years were asked about school attendance. All individuals examined were asked whether they had taken azithromycin during previous MDA campaigns; examiners used containers of azithromycin tablets as visual aids. One adult in each household was questioned about the location of the water source used for daily household tasks. Latrines in households were visually inspected to confirm use and type (modern or traditional). GPS coordinate data were gathered for each cluster.

### Training of surveyors and survey teams

Prior to the implementation of the surveys, the PNSO, with support from technical and financial partners, trained examiners and interviewers. The training followed the guidelines of the Global Trachoma Mapping Project (GTMP) [24]. At the end of the training, examiners were given post-training tests to assess their skills. A Cohen’s kappa coefficient was calculated according to their test scores, using the excel tool developed by the GTMP [24]. Only those with a kappa value of ≥0.80 were chosen to participate in the implementation of the surveys. In addition to the initial training, the examiners received refresher training on screening for clinical signs of trachoma prior to each survey. Several tools, such as standard survey procedures, supervision check list, images of trachoma signs at different stages, etc., were also developed and provided to the survey teams.

### Data management and analysis

Data were collected using the ONA interface on tablet computers and analyzed using Excel (version 2003, Microsoft, Seattle, US), Epi Info 7.2.1.0 (CDC, Atlanta, US) and R software (version 2.15.2, R Foundation for Statistical Computing, Vienna, Austria). Fisher’s exact test and Pearson χ^2^ were used to compare proportions between gender and signs of trachoma and between the SAFE strategy components and signs of trachoma. A 95% confidence interval (CI) was calculated for prevalence. TT backlog was calculated taking the TT prevalence and the district population into account. A Z-test was used to compare trachoma prevalence from the present study with prevalence in 2009 [15].

### Ethical approval

The survey was conducted as part of routine monitoring and evaluation activities of the national program of the Ministry of Health to eliminate trachoma as a public health problem in Mali. As it was a standard public health measure and all procedures for survey and diagnosis were following the WHO guidelines, review of the protocol by the Institutional Review Board was considered not necessary and therefore not conducted. Authorization was sought, and granted, from village chiefs before surveys were conducted in the selected villages. At the household level, oral informed consent was obtained from the heads of the selected households, and any other adult, parent or caregiver, prior to beginning the survey for their participation and that of their children. In addition, assent was obtained from each child >6 years who participated. Survey participants were informed of the purpose of the trachoma examinations and their rights not to participate or to stop the examination at any time. Oral consent was documented on a survey electronic data collection tool. All children presenting signs of TF or trachomatous inflammation—intense (TI) were provided free tetracycline eye ointment and caregivers were instructed to apply it twice daily for six weeks. Those identified with TT were recorded, counselled, and offered free consultation and surgery with a trained TT surgeon.

## Results

A total of 14,159 people aged ≥1 year were enumerated and 11,620 (82.1%) were examined in the selected households in the health districts of Kayes, Kéniéba, Nioro and Yélimané. Of those examined, 4,502 (38.7%) were children aged 1–9 years and 5,573 (48%) were aged 15 years and above. Table 1 shows the details by district. Females represented 57.1% of the total number of people examined.

**Table 1:** Demographic information (2015), clusters and number of persons examined in each district.

Districts	No. health areas	No. villages	2015 population	Number of clusters	Total eligible	Total examined N (%)	1–9 years examined n (%)	10–14 years examined n (%)	≥15 years examined n (%)	Male examined n (%)	Female examined n (%)
Kayes	43	398	628,587	40	5,605	4,484 (80.0)	1,687 (37.6)	595 (13.3)	2,202 (49.1)	1,883 (42.0)	2,601 (58.0)
Kéniéba	22	206	237,731	20	2,945	2,383 (80.9)	990 (41.5)	309 (13.0)	1,084 (45.5)	1,052 (44.1)	1,331 (55.9)
Nioro	24	196	282,221	20	2,595	2,161 (83.3)	860 (39.8)	240 (11.1)	1,061 (49.1)	941 (43.5)	1,220 (56.5)
Yélimané	27	92	218,494	20	3,014	2,592 (86.0)	965 (37.2)	401 (15.1)	1,226 (47.3)	1,108 (42.7)	1,484 (57.3)
Total	218	1,599	2,445,000	100	14,159	11,620 (82.1)	4,502 (38.7)	1,545 (13.3)	5,573 (48.0)	4,984 (42.9)	6,636 (57.1)

### Prevalence of TF in children aged 1–9 years

The TF prevalence was <5% in all four health districts surveyed, ranging from 0.23% to 0.81%. The prevalence and 95% confidence interval was 0.53% (95% CI: 0.28–1.01%); 0.81% (95% CI: 0.41–1.59%); 0.23% (95% CI: 0.06–0.84%) and 0.31% (95% CI: 0.11–0.91%) for the districts of Kayes, Kéniéba, Nioro and Yélimané, respectively (Table 2). Boys 1–9 years had less than half the prevalence of TF compared to girls of the same age, however the difference was not statistically significant (Pearson χ^2^, p>0.05) (Table 2). In contrast to the survey conducted in 2009, all four districts reached the TF elimination threshold and the difference between TF prevalence in 2009 and 2015 was statistically significant for all districts (p<0.01; Table 3).

**Table 2:** Prevalence of active trachoma (TF) and trichiasis (TT) in four districts in Kayes region in 2015.

	TF % in 1–9 years (95% CI)^a^	TT % in all ages (95% CI)^b^	TT % in ≥15 years (95% CI)^c^
By districts			
Kayes	0.53 (0.28–1.01)	0.06 (0.01–0.19)	0.09 (0.02–0.33)
Kéniéba	0.81 (0.41–1.59)	0.29 (0.11–0.60)	0.65 (0.31–1.33)
Nioro	0.23 (0.06–0.84)	0.04 (0.00–0.25)	0.09 (0.02–0.53)
Yélimané	0.31 (0.11–0.91)	0.07 (0.00–0.27)	0.16 (0.04–0.59)
By sex^d^			
Male	0.31 (0.15–0.63)	0.16 (0.08–0.32)	0.36 (0.17–0.47)
Female	0.68 (0.41–1.12)	0.08 (0.03–0.18)	0.14 (0.06–0.33)
Total	0.49 (0.32–0.74)	0.11 (0.05–0.19)	0.22 (0.12–0.38)

Note:

^a^ Fisher test, p = 0.3 among districts;

^b^ Fisher test, p = 0.04 among districts;

^c^ Fisher test, p = 0.02 among districts

^d^ Pearson χ^2^ all p>0.05 between male and female.

**Table 3:** Comparison of trachoma TF and TT prevalence between 2009 and 2015.

Health district	Prevalence (%) in 2009 (95% CI)^a^	Prevalence (%) in 2015 (95% CI)	Z value	Prevalence difference 2009–2015 (95% CI)	P value
**TF prevalence in children (1–9 years)**					
Kayes	5.3 (3.4–7.2)	0.5 (0.3–1)	4.87	4.8 (2.83–6.77)	<0.01
Kéniéba	7.1 (3.5–10.8)	0.8 (0.4–1.7)	3.38	6.3 (2.57–10.03)	<0.01
Nioro	8.7 (6.0–11.3)	0.2 (0–0.9)	6.20	8.5 (5.76–11.24)	<0.01
Yélimané	6.5 (4.8–8.1)	0.3 (0.1–1)	7.11	6.2 (4.45–7.95)	<0.01
**TT prevalence (%) in all ages ≥1 year**					
Kayes	0.75 (0.42–1.08)	0.06 (0.01–0.19)	4.06	0.69 (0.35–1.03)	<0.001
Kéniéba	0.78 (0.38–1.16)	0.29 (0.11–0.60)	2.09	0.49 (0.02–0.96)	0.018
Nioro	0.59 (0.09–1.09)	0.04 (0.00–0.25)	2.09	0.55 (0.02–1.08)	0.019
Yélimané	0.20 (0.0–0.45)	0.07 (0.00–0.27)	0.97	0.13 (-0.14–0.40)	0.16

Note:

^a^ Data from previous publication [15].

### Prevalence of TT

The prevalence of unknown TT cases in the population examined (≥1 year old) was less than 0.1% in three of the four surveyed districts. The prevalence was 0.04% (95% CI: 0.00–0.25%) in Kayes, 0.04% (95% CI: 0.00–0.25%) in Nioro and 0.07% (95% CI: 0.00–0.27%) in Yélimané. The TT prevalence in Kéniéba was 0.29% (95% CI: 0.11–0.60%), which is greater than the WHO elimination threshold (>0.1%). Although five (38.5%) of the 13 TT patients found were female, the difference in TT prevalence between males and females was not statically significant (data not shown, p>0.05). TT prevalence decreased considerably from 2009 to 2015 for all districts (p<0.05, except Yélimané district p = 0.16) (Table 3).

The prevalence of TT in those aged ≥15 years ranged from 0.09% to 0.65% in the four districts surveyed (Table 2). The prevalence was 0.09% (95%, CI: 0.02–0.33%) in Kayes; 0.65% (95%, CI: 0.31–1.33%) in Kéniéba, 0.09% (95%, CI: 0.02–0.53%) in Nioro, and 0.16% (95%, CI: 0.04–0.59%) in Yélimané (Table 2).

Based on the 2015 survey, the TT backlog i.e. those patients who needed surgery but had not yet received it, was recalculated from 2009 to 2015 respectively to: 3,850 to 314 in Kayes; 1,495 to 571 in Kéniéba; 360 to 113 in Nioro and 357 to 131 in Yélimané.

### Facial cleanliness, sanitation and water sources

More than 85% of children aged 1–9 years had a clean face at the time of the survey. This proportion was 92.1% in Kayes, 81.1% in Kéniéba, 88.5% in Nioro, 78.2% in Yélimané (Table 4). Basic sanitation (household with a latrine) was evident in 89.5% (range: 70.9–96.8%) of the households of the four districts (Table 4). A water source inside the household compound was observed in 21.3% (range: 9.3–36.9%) of surveyed households and 6% (range: 0–13.5%) of the surveyed households reported having to travel outside the geographical boundaries of the village to collect water (Table 4). The proportion of households with a water source inside the household compound was very low in the district of Kéniéba (9.3%) and Nioro (10.6%). We observed that 100% of surveyed households had their water source inside of their house or inside the village in Yélimané while in Kéniéba 13.5% went outside the village to find water.

**Table 4:** Clean faces and access to latrines and water sources in the surveyed clusters in Kayes region in 2015.

Districts	No of household surveyed	No of household with a latrine (%)	No of household with access to water source			No of children (1–9 years) surveyed	No of children (1–9 years) with clean face
			Within household compound (%)	Within village (%)	Outside village (%)		
Kayes	1144	1108 (96.9)	282 (24.7)	831 (72.6)	31 (2.7)	1,687	1,553 (92.1)
Kéniéba	547	388 (70.9)	51 (9.3)	422 (77.1)	74 (13.5)	990	803 (81.1)
Nioro	557	475 (85.9)	59 (10.6)	434 (77.9)	64 (11.5)	860	761 (88.5)
Yélimané	561	543 (96.8)	207 (36.9)	354 (63.1)	0 (0)	965	755 (78.2)
Total	**2809**	2514 (89.5)	599 (21.3)	2041 (72.7)	169 (6)	4,502	3,872 (86)

Among the 4,502 children (1–9 years) examined, those with clean faces had a TF prevalence of less than one-half that of children who did not have clean faces (0.41% vs 0.95%, p = 0.07, Table 5). Among children in households having a water source within household compound, the TF prevalence was 0.43% which was not statistically different from 0.51% in other children (Fisher, p = 0.50, Table 5). Similarly, among adults (≥15 years) in households having water source within household compound, the TT prevalence was 0.16% which was similar to 0.23% in adults without water source within household compound (Fisher, p = 0.45, Table 5).

**Table 5:** Trachoma signs and risk factors.

	**TF among children aged 1–9 years**		**Fisher Exact Test**
	**No**	**Yes**	
**Clean face**			
No (n = 630)	624 (99.05%)	6 (0.95%)	p = 0.07
Yes (n = 3872)	3,856 (99.59%)	16 (0.41%)	
**Water source**			
Within household Compound (n = 938)	934 (99.57%)	4 (0.43%)	p = 0.50
Outside household compound (n = 3564)	3,546 (99.49%)	18 (0.51%)	
	**TT among people over 15 years**		**Fisher Exact Test**
	**No**	**Yes**	
**Water source**			
Within household compound (n = 1274)	1,272 (99.84%)	2 (0.16%)	p = 0.45
Outside household compound (n = 4299)	4,289 (99.77%)	10 (0.23%)	

## Discussion

Eight years after stopping MDA while intensifying efforts to provide trichiasis surgery, four districts in the Kayes region of Mali reached the TF elimination threshold of <5% and three of four districts reached the TT elimination threshold of <0.1%. Between 2004–2006, the Kayes, Kéniéba, Nioro and Yélimané health districts benefited from annual MDA with azithromycin and 1% tetracycline eye ointment accompanied by intensified trichiasis surgery campaigns from 2001 to 2015. However, no MDA was conducted in health districts since the last MDA in 2006. Although the 2009 survey data suggested that further sub-district level MDA was needed, according to the WHO 2006 guidelines [14], the PNSO opted not to pursue this route and instead enhanced S, F and E activities in the districts. The result of this approach was evident with more than 78% (ranging from 78.2% to 92.1%) of children observed with clean faces and 94% of villages with water sources inside the household or in the village. TF prevalence among children aged 1–9 years in 2015 fell significantly from that in 2009 for each district.

There was no significant difference in terms of methodology used in 2009 and in 2015. Changes in 2015 however included the use of tablet computers by interviewers and supervisors and the confirmation of all TF and TT cases by supervisors. These steps could be expected to increase data accuracy. A similar situation was noted in Kidal, a region of Mali where TF prevalence fell from 46.2% to 15.6% over 12 years without MDA [25], and in other African countries where TF prevalence declined dramatically without antibiotic intervention [26, 27]. In the Gambia, one round of treatment achieved long-term and sustained reduction of trachoma [28]. Such reduction was attributed to the improvement in development and environmental conditions [26–28]. The significant reduction in TF prevalence in these districts surveyed without resuming MDA may have been due to the similar reasons and to the influence of ongoing activities of other SAFE components. There was an increase in implementing activities related to TT surgery which focused on reducing the TT backlog. The facial cleanliness and environmental improvement components of the SAFE strategy were also implemented. Awareness messages were broadcast on radio stations and women’s groups and community health workers received training to discuss trachoma and prevention strategies. Combined, it is likely that these additional interventions contributed to the reduction in TF in the absence of MDA. This was reflected by the data on clean faces, use of latrines and availability of water source with household compounds.

TF prevalence was less than half as common among boys as girls, although this difference was not statistically significant as our study was not powered to detect this difference (Table 2). Among children with TF, having an unclean face was twice as likely as a clean face. Again, small numbers likely contributed to this result not reaching the threshold of statistical significance (p = 0.07). A significant association did not exist between the lack of a water source within their household compound and the presence of TF (p>0.05, Table 5). The presence of follicular trachoma is much more closely associated with living conditions (poverty, poor hygiene, and lack of water or access to water) [29]. It should also be noted that many villages in the Kayes, Nioro and Yélimané health districts have a clean water source within the residential cluster. The villages in these districts received considerable support from emigrants living in the West, who contributed money to build latrines, health infrastructure, and water infrastructures. The Kéniéba health district had not enjoyed similar benefits due to the poor geographical accessibility of villages and high poverty level of communities in the district.

The interventions carried out in these districts—specifically, regular mobile surgery campaigns—may explain this significant reduction in TT prevalence in all four health districts. This strategy involves screening and operating on TT patients on a village-by-village basis. Surgery is performed by a TT surgeon traveling by motorcycle or a team of surgeons traveling by car. In addition to the mobile strategy, patients also had surgery at fixed sites in referral health centers and at the district hospital in Kayes. However, in Kéniéba district which had not reached the TT elimination criterion, there are a large number of gold mines that attract migrant workers. These migrant workers are more likely to refuse TT surgery, considering this a distraction from gold panning (an observation by authors during program supervision, detail not shown). In addition, these gold mine sites were sometimes not visited by the surgical teams because they are not official villages and thus not counted during screening. The TT surgical refusal rate was higher than 20% sometimes according the TT surgery activity reports in this district. This may explain the small drop in TT prevalence, despite the good geographical coverage of health areas for surgical campaign.

Improved hygiene and sanitation play an important role in controlling the transmission of trachoma [30]. Facial cleanliness and environmental improvement activities have been limited and often uncoordinated in the past. Women’s groups in these districts held educational talks with their peers and gathered to listen to the trachoma radio broadcasts together. Many non-governmental organizations are also involved in WASH activities that contribute, directly or indirectly, to reducing the prevalence of active trachoma. The proportion of children aged 1–9 years with clean faces varied among the health districts, ranging from 78.2% to 92.1%. Accessibility to a latrine (having a latrine in the household) ranged from 69.3% in Kéniéba to 97.3% in Yélimané (Table 4). According to a 2009 survey in several countries, Mali’s overall latrine coverage exceeded 90% [31], and according to the Demography and Health survey conducted in Mali in 2012 and 2013, the households without any kind of latrine represented 11%, which varied from 14% for rural households to 1% in urban areas [32]. The water source was located primarily either within the household compound or in the village. The percentage of those surveyed whose water source was outside the village ranged from 0% in Yélimané to 13.6% in Kéniéba. These variations may be explained by the level of development of the villages in the districts surveyed, particularly Kayes and Yélimané districts. The F&E data showed that there was a need to reinforce latrine accessibility in Kéniéba (69.3%) and water sources inside of households for all four HDs (average 22.7%) even though the number of villages with a water source inside the village was relatively high at 71.3%. Improvement in these areas may have all contributed to the continued decline of trachoma prevalence without further MDA in these districts [33–36].

One limitation of this study was that the current survey was not powered to give an accurate estimate of TT prevalence. This underpowering may have contributed to the large spread of the 95% CI of the TT and TF prevalence. Despite that TT prevalence in three districts met the elimination threshold, it is noted that the upper bound of the 95% CI of TT prevalence in these districts was above the 0.1% elimination threshold in the population. Secondly, the result on TT in the 2015 survey was recorded differently from the 2009 survey as only those cases unknown to health systems were counted as TT cases in the result. The 2015 TT result was not fully comparable to the 2009 result; however, it did represent the field situation at the time. Based on the current data, the national program should continue with the TT surgery effort in all four districts for the remaining TT cases. For future trachoma survey to verify whether the TT prevalence in an EU has reached the elimination threshold, a properly powered TT survey may be needed.

In conclusion, these results showed that the TF prevalence in children aged 1 to 9 years fell considerably between 2009 and 2015 for all four HDs without the need of one additional round of MDA. All four health districts met the TF elimination criteria and three of the four surveyed met the TT elimination threshold. Based on these results, the TT surgery campaigns will continue only in the Kéniéba health district. The PNSO also recommended that in Kayes, Nioro and Yélimané districts, TT case management should continue in fixed-site centers in hospitals and the referral health centers in order to ensure availability of surgery for remaining TT cases after elimination of trachoma as a public health problem has been achieved. The results of these surveys show that Mali is on the right track toward eliminating trachoma as a public health problem by 2018.

## Supporting information

S1 Checklist STROBE checklist. (DOC)

S1 Dataset Database of trachoma impact survey in four health districts of Kayes region, Mali in 2015. (XLS)
